# High intensity focused ultrasound enhances anti-tumor immunity by inhibiting the negative regulatory effect of miR-134 on CD86 in a murine melanoma model

**DOI:** 10.18632/oncotarget.5285

**Published:** 2015-10-16

**Authors:** Shi-Mei Yuan, Huan Li, Min Yang, He Zha, Hui Sun, Xue-Ru Li, Ai-Fang Li, Yue Gu, Liang Duan, Jin-Yong Luo, Chong-Yan Li, Yan Wang, Zhi-Biao Wang, Tong-Chuan He, Lan Zhou

**Affiliations:** ^1^ Key Laboratory of Clinical Diagnosis of Education Ministry, College of Laboratory Medicine, Chongqing Medical University, Chongqing 400016, China; ^2^ State Key Laboratory of Ultrasound Engineering in Medicine Co-founded by Chongqing and the Ministry of Science and Technology, Chongqing Key Laboratory of Ultrasound in Medicine and Engineering, College of Biomedical Engineering, Chongqing Medical University, Chongqing 400016, China; ^3^ Molecular Oncology Laboratory, Department of Orthopaedic Surgery and Rehabilitation Medicine, The University of Chicago Medical Center, Chicago, IL 60637, USA

**Keywords:** HIFU, anti-tumor immunity, microRNA, co-stimulatory molecules

## Abstract

HIFU has been demonstrated to enhance anti-tumor immunity, however, the mechanism of which has not been well elucidated. Emerging evidence indicates that miRNAs play important roles in immune response. In this study, we used the B16F10 melanoma allograft mouse model to investigate the role of miRNAs in HIFU-enhanced anti-tumor immunity. We found that HIFU treatment decreased circulating B16F10 cells and pulmonary metastasis nodules while increased IFN-γ and TNF-α in the peripheral blood and cumulative mouse survival, which was associated with inhibition of miR-134 expression and activation of CD86 expression in tumor tissues. Further, we determined that miR-134 directly binds to the 3′UTR of CD86 mRNA to suppress its expression in B16F10 cells. When B16F10 cells transfected with miR-134 were co-cultured with normal splenic lymphocytes, the secretion of IFN-γ and TNF-α from lymphocytes was reduced and B16F10 cell survival was increased. HIFU exposure efficiently decreased miR-134 while increased CD86 expression in B16F10 cells *in vitro*. CD86 knockdown with siRNA markedly rescued the viability of HIFU-treated B16F10 cells that co-cultured with lymphocytes. Altogether, our results suggest that HIFU down-regulates miR-134 to release the inhibition of miR-134 on CD86 in melanoma cells, thereby enhancing anti-tumor immune response.

## INTRODUCTION

High-intensity focused ultrasound (HIFU) is an emerging non-invasive ablation technique that takes advantage of the ultrasonic wave to target tumor entities, causing protein degeneration and coagulative necrosis of tumor tissue. HIFU has been shown to improve the prognosis of patients with malignant tumors such as pancreatic and prostate cancer and melanoma [1, 2]. In the past few years, it was found that HIFU not only eradicates primary tumors, inhibits tumor relapse and distant metastasis, it also triggers systemic anti-tumor immunity [3, 4]. There are different views and hypotheses about the mechanism of HIFU-evoked anti-tumor immunity. For example, HIFU may effectively decrease tumor loads to restore immune system function [5]. HIFU-induced oncolysis may lead to the release of tumor-associated antigens [6, 7]. HIFU also induces the release of heat shock protein to stimulate the host immune system [8]. However, the precise mechanisms are still not fully elucidated.

Host anti-tumor immunity is primarily mediated by T lymphocytes that are activated by a series of signals for appropriate antigen presentation to the TCR (signal 1) and a second co-stimulatory signal (signal 2) [9]. Numerous studies have found that lack of co-stimulatory molecules on the membrane of antigen-presenting cells (APC) or tumor cell is an important cause of immune tolerance in cancer patients [10–11]. miRNAs play important roles in T lymphocytes differentiation, maturation, immune response and immune tolerance [12, 13], through binding to the 3′-untranslated regions (3′UTR) of target genes to suppress their expression. Therefore, we reasoned that HIFU may enhance the anti-tumor immunity by modulating the expression and function of miRNAs in tumor cells.

In this study, we established a B16F10 melanoma allograft mouse model for investigating the mechanism by which HIFU modulates anti-tumor immunity. After confirming that HIFU can enhance anti-tumor immunity, we focused on differential expression of miRNAs in tumors after HIFU treatment and searched the target genes of these miRNAs with a bioinformatics approach and a signal cascade consists of HIFU, miR-134 and CD86 was identified. The results suggest that HIFU down-regulates miR-134 to release the inhibition of miR-134 on CD86 in melanoma cells, thereby enhancing anti-tumor immune response. This signaling cascade may be exploited for clinical application of HIFU for melanoma therapy.

## RESULTS

### HIFU damaged melanoma tumor tissue, inhibited tumor growth and distant metastasis, and improved mouse survival

**Figure 1:**
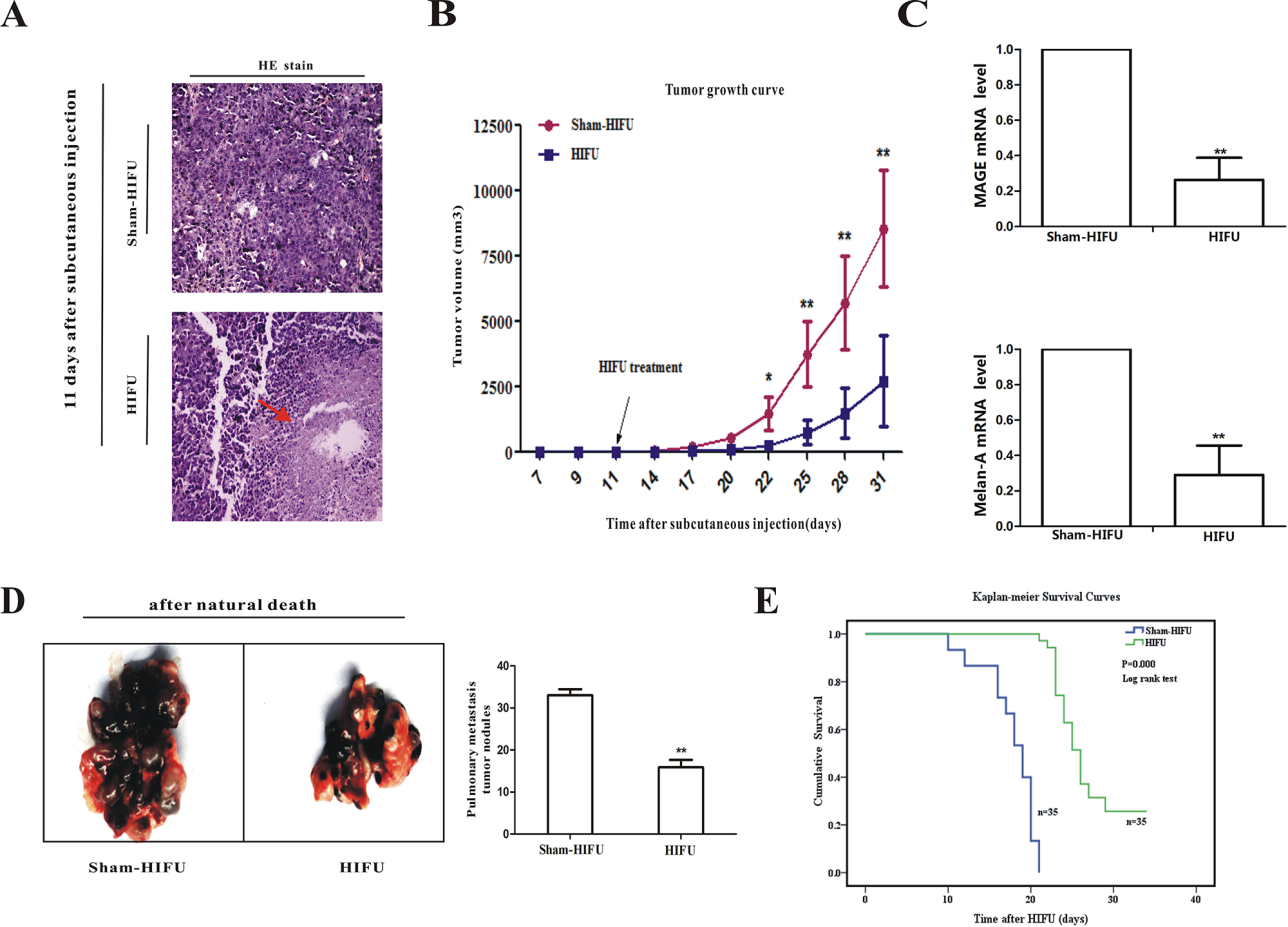
HIFU treatment had a melanoma suppressing effect **A.** The tumor tissues were taken and HE staining after subcutaneous injection 11 days (immediately after HIFU treatment; original magnification, × 200). **B.** The average tumor volumes of HIFU and sham-HIFU mice were plotted as a function of time in days. **C.** qPCR was used to detect the levels of MAGE and Melan-A in the peripheral blood at 14^th^ day after HIFU treatment. Data shown are mean ± SD. **D.** Pulmonary metastasis tumor nodules were counted macroscopically after natural death. **E.** The cumulative survival rate was analyzed by log rank test. *p < 0.05, **p < 0.01, when compared to sham-HIFU mice.

When the large diameter of tumors reached 7–8 mm (about 11 days after subcutaneous injection), the tumor-bearing mice were randomly divided into two groups and treated with HIFU or a sham-HIFU procedure. As shown in Figure 1A, HIFU treatment caused coagulation necrosis in the focused zone immediately (indicated by red arrow), while having no obvious effect on the surrounding tissues. There was a clear separation in growth curves of the residual primary tumor. The primary tumor volume in the HIFU group was significantly smaller than that of the sham-HIFU group, starting from 22 days after subcutaneous cell injection (11 days after HIFU treatment) to the end of study (Figure 1B). At day 14 after HIFU treatment, the median volume of tumor tissues in the HIFU group was 416 mm^3^, which was significantly smaller than that of the sham-HIFU group, 3600 mm^3^ (*p* < 0.01).

For studying the effect of HIFU on metastasis, the mice with primary tumor were injected with B16F10 melanoma cells via the tail veins seven days after HIFU treatment. Because circulating tumor cells (CTC) presenting in the peripheral blood is a prerequisite step of distant metastases [14], we examined CTC in the animals at 14^th^ day after HIFU treatment by detecting the mRNA of melanocytic markers melanoma antigen gene (MAGE) and Melan-A by qPCR [15–19]. MAGE and Melan-A were significantly reduced in the peripheral blood of mice after HIFU treatment (Figure 1C). When the mice died a nature death, the pulmonary metastasis tumor nodule number in the HIFU group was significantly lower than that in the sham-HIFU group (*p* < 0.01, Figure 1D). The cumulative survival rate of HIFU-treated mice was statistically higher than that of the control (*p* < 0.01, Figure 1E). Altogether, these experiments show that HIFU could suppress tumor growth and distant metastasis, and improve host survival, suggesting that HIFU treatment could be a good choice for melanoma therapy.

### HIFU treatment enhanced anti-tumor immune response

**Figure 2:**
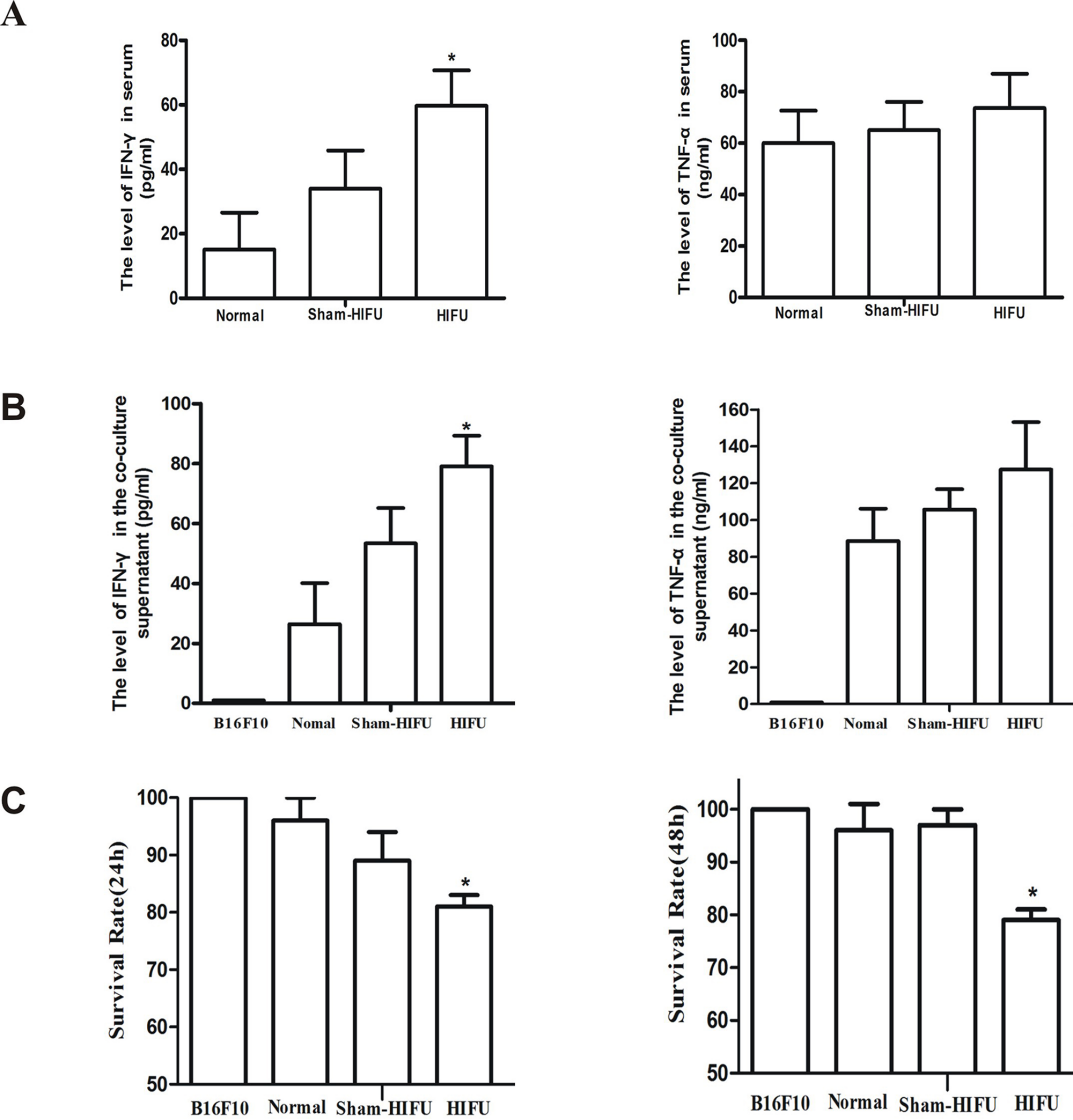
HIFU treatment enhanced anti-tumor immune response **A.** IFN-γ and TNF-α in serum of mice were analyzed by ELISA. **B.** Purified splenic lymphocytes from each group were co-cultured with B16F10 cells *in vitro*, and then the levels of IFN-γ and TNF-α in co-culture supernatant were analyzed using specific ELISA kits. **C.** The cytotoxic activity of lymphocytes against B16F10 cells was measured by MTT. Results are expressed as the mean ± SD, and the values are from at least three independent experiments. *p < 0.05, **p < 0.01 as compared with sham-HIFU.

The mean serum level of IFN-γ in the HIFU group was 60 pg/ml, which was significantly higher than that in the normal group (15 pg/ml) and sham-HIFU group (33 pg/ml). The serum level of TNF-α showed a stably increasing trend after HIFU treatment. However, the trend did not reach a statistical significance (*p* > 0.05) (Figure 2A). These results were consistent with literature that HIFU may promote anticancer immunity through modulating cytokine secretion [20, 21].

For elucidating the mechanism of HIFU in modulating anticancer immunity, splenic lymphocytes from each group were co-cultured with B16F10 cells *in vitro*. Consistent with the results of mouse serum, the mean level of IFN-γ in co-culture supernatants from HIFU group was 79 pg/ml, which was significantly higher than that from B16F10 only (1 pg/ml), normal (26 pg/ml) and sham-HIFU groups (53 pg/ml). TNF-α level also showed a steady increase trend in the HIFU-treat group (Figure 2B). The survival rates of B16F10 cells in B16F10 only, normal, sham-HIFU, and HIFU groups were 100%, 96%, 89%, and 81% at 24 h and were 100%, 96%, 97%, and 79% at 48 h, respectively (*p* < 0.05, Figure 2C). These results suggest that HIFU is able to enhance the lymphocyte-mediated killing of B16F10 cells, which may involve IFN-γ and TNF-α secretion from lymphocytes.

### HIFU treatment caused differential miRNA expression in tumor tissue

**Figure 3:**
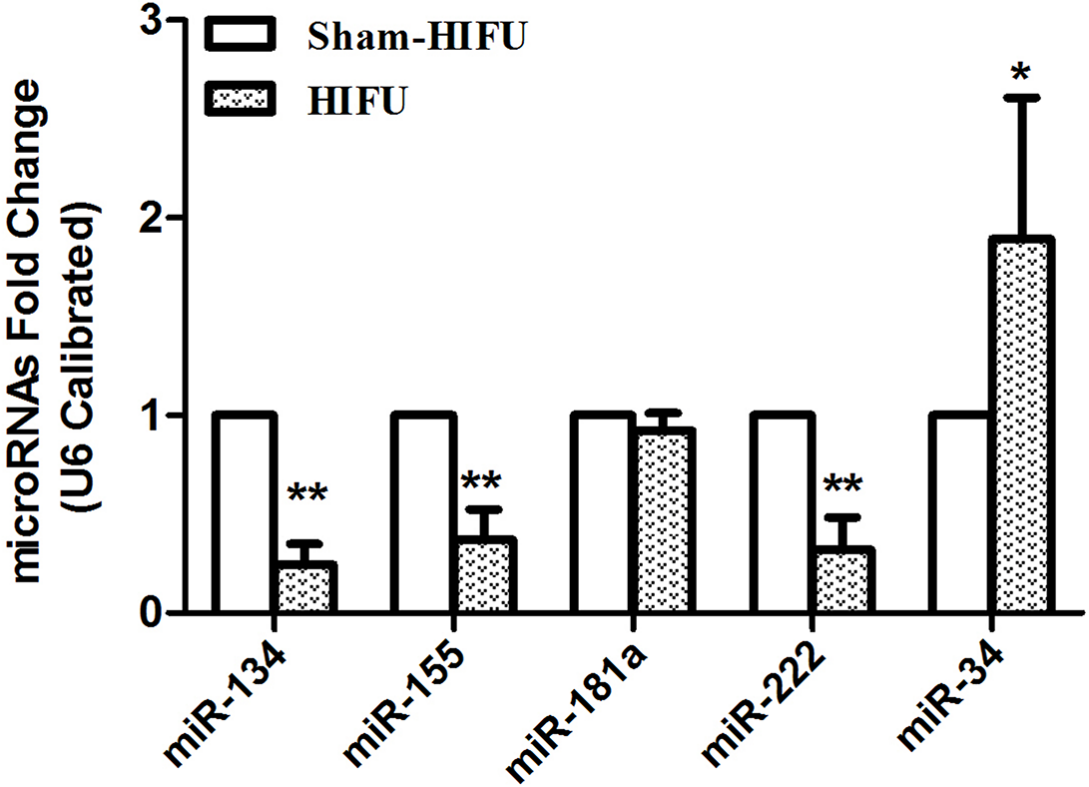
HIFU treatment caused the differential expressions of tumor tissue's miRNAs The levels of miRNAs in tumor tissues were detected by qPCR (U6 as an internal control). Data are represented as means ± SD. *p ≤ 0.05, **p ≤ 0.01 compared to sham-HIFU group.

Previous studies have demonstrated that HIFU can enhance the anti-tumor immunity [22, 23]; however, the mechanism of which is not well elucidated. Given that miRNAs are involved in immune response, we sought to investigate whether miRNAs participate in HIFU-enhanced anti-tumor immune response. Eight miRNAs that are closely associated with immune response reported in literatures were examined by qPCR [12, 13]. These included miR-34, miR-106a, miR-126a, miR-134, miR-155, miR-181a, miR-221, and miR-222. The results showed that miR-134, miR-155 and miR-222 were down-regulated while miR-34 was up-regulated in the HIFU group (Figure 3). The expression levels of miR-106a, miR-126a and miR-221 were very low in tumor tissues and there were no difference between the HIFU and sham-HIFU groups (Data not shown).

### HIFU increased the expressions of CD86 and ICAM-1 in tumor tissue

**Figure 4:**
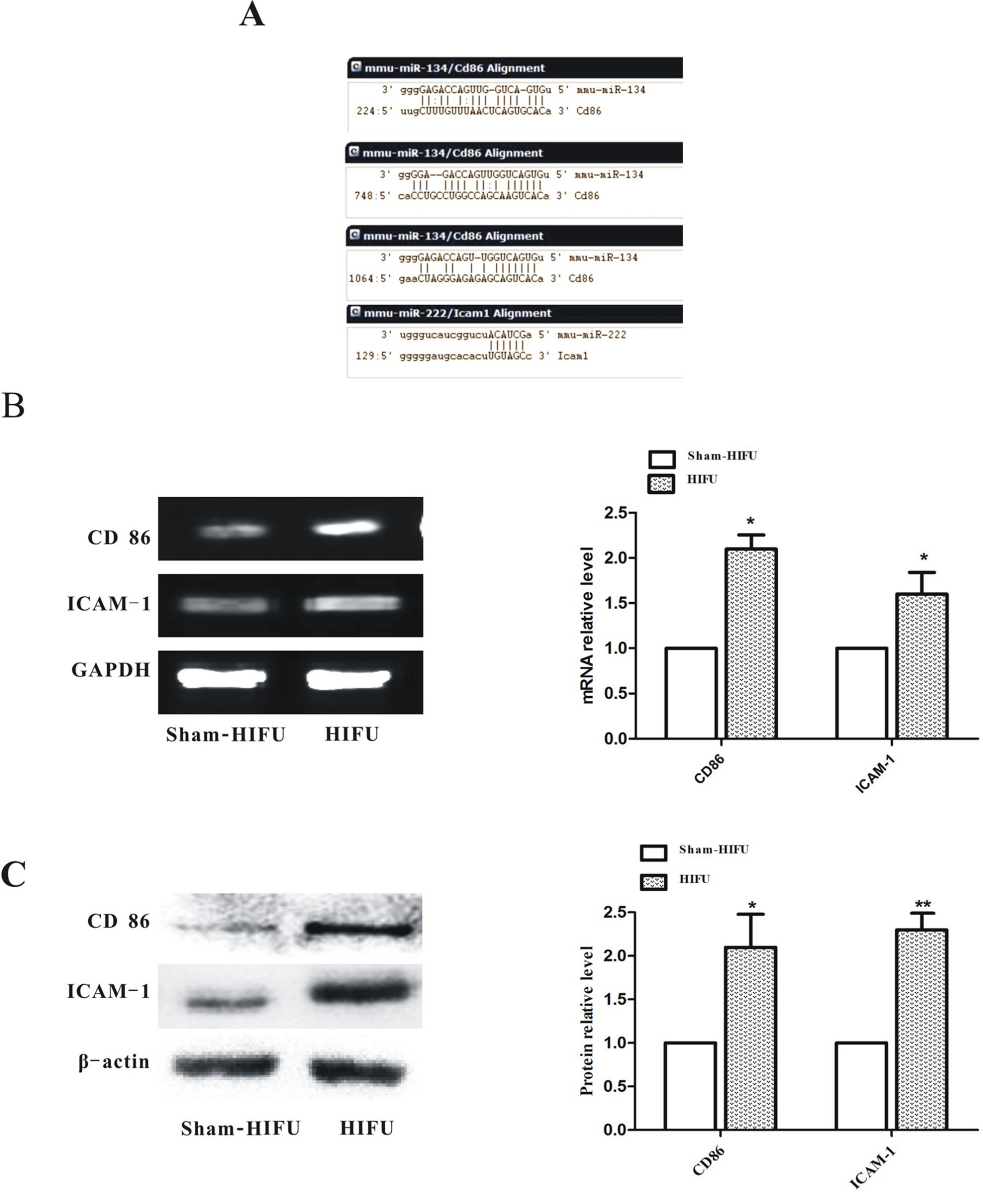
HIFU treatment increased the levels of CD86 and ICAM-1 in tumor tissue **A.** The target sites of miRNAs were predicted by bioinformatics analysis. **B.** The mRNA levels of CD86, ICAM-1 and GAPDH were detected by Semi-quantitative PCR. **C.** The protein levels of CD86, ICAM-1 and β-actin in tumor tissue were determined by Western blot. *p ≤ 0.05,**p ≤ 0.01 compared to sham-HIFU group.

Three target prediction tools were used to predict the potential target genes of the differentially expressed miRNAs. Among the target genes that were predicted by at least two tools, co-stimulatory molecules CD86 and ICAM-1 that are involved in T cell activation were focused [24–26]. As shown in Figure 4A, there were three miR-134 binding sites in CD86 3′UTR and one miR-222 binding site in ICAM-1 3′UTR. The mRNA levels of CD86 and ICAM-1 in tumor tissues collected at 14^th^ day after HIFU treatment were significantly increased in the HIFU group (Figure 4B). The protein levels of CD86 and ICAM-1 in the HIFU group were 2.1-fold (*p* < 0.05) and 2.3-fold (*p* < 0.01) of the sham-HIFU group, respectively (Figure 4C). Altogether, these results suggest that HIFU increases the expressions of CD86 and ICAM-1 in melanoma tumor likely through miRNA.

### miR-134 directly bound to CD86 mRNA 3′UTR to suppress CD86 expression

**Figure 5:**
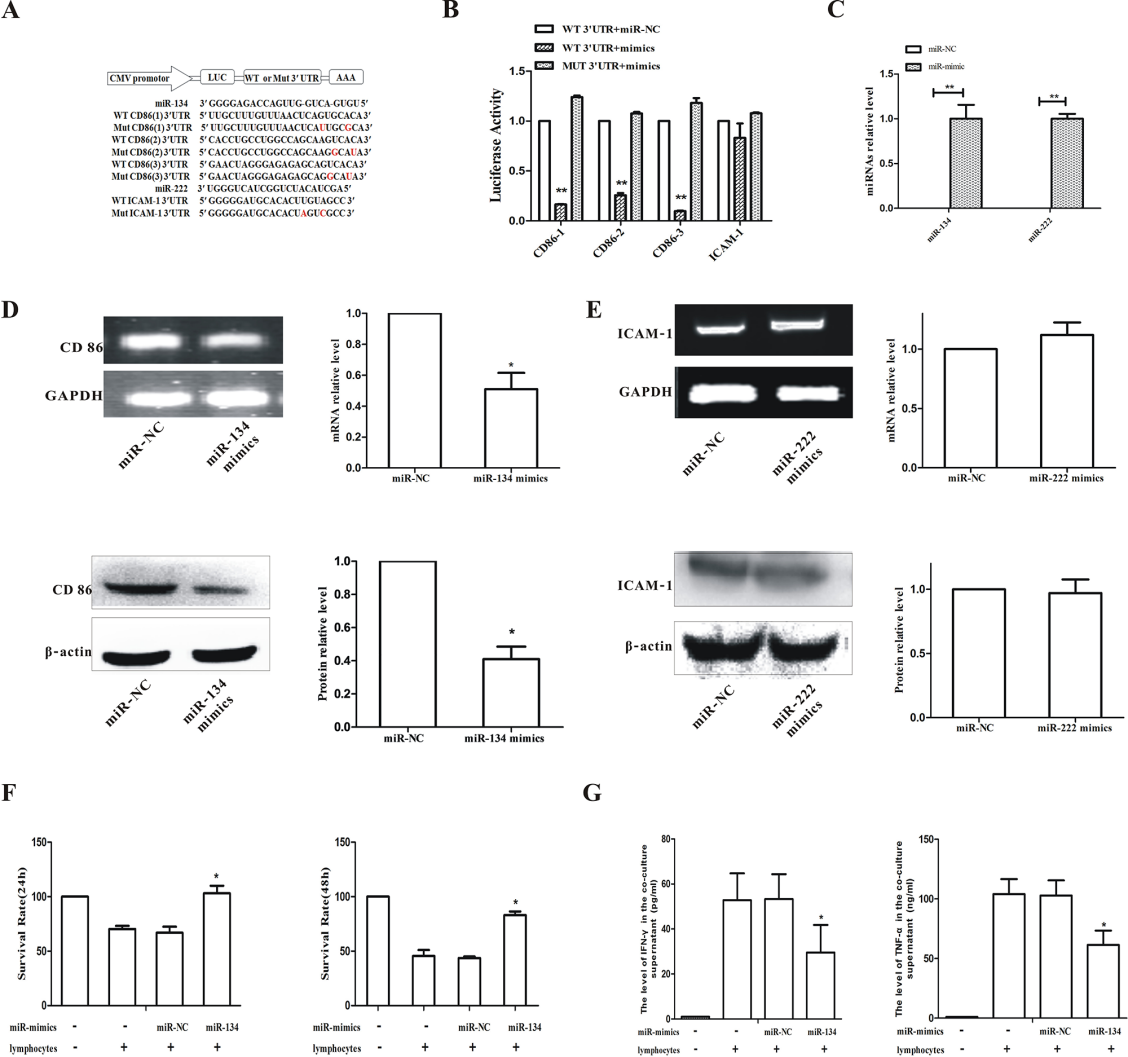
miR-134 directly bound to CD86 mRNA 3′UTR to suppress CD86 expression in B16F10 cells and the cytotoxic activity of lymphocytes **A.** Schematic diagram was used to illustrate the design of luciferase reporters with the wild-type (WT 3′UTR) or the site-directed mutant 3′UTR (Mut 3′UTR). **B.** The effect of miR-NC and miR-mimics on luciferase activity in B16F10 cells transfected with either the WT 3′UTR reporter or the mutant 3′UTR reporter. Firefly and renilla luciferases were measured in cell lysate. **C.** B16F10 cells were transfected with miR-134 mimics or miR-222 mimics for 48 h and the miRNA levels of miR-134 and miR-222 were determined by qPCR. **D.** B16F10 cells were transfected with miR-134 mimics for 48 h and the mRNA (upper panel) and protein (lower panel) levels of CD86 were determined. **E.** B16F10 cells were transfected with miR-222 mimics for 48 h and the mRNA (upper panel) and protein (lower panel) expression of ICAM-1 was detected. **F.** B16F10 cells transfected with miR-134 were co-cultured with B16F10-activated lymphocytes, and the cytotoxic of lymphocytes against B16F10 cells was measured by MTT. **G.** IFN-γ (left panel) and TNF-α (right panel) in co-culture supernatant was analyzed by ELISA. *p ≤ 0.05, **p ≤ 0.01 compared to that of miR-NC.

To determine whether miR-134 directly binds to the 3′UTR of CD86 mRNA and miR-222 binds to ICAM-1 mRNA, luciferase reporters that carry either the wild-type (WT) 3′UTR sequences or the mutant (MUT) 3′UTR sequences of the CD86 (for miR-134) or ICAM-1 (for miR-222) were constructed (Figure 5A), and co-transfected into B16F10 cells with or without miRNAs mimics. The luciferase activity of the luciferase reporters containing WT but not the mutated 3′UTR of CD86 was significantly inhibited by miR-134 mimics (*p* < 0.01, Figure 5B). In contrast, the luciferase reporter activity of the reporters bearing either WT or mutated 3′UTR of ICAM-1 was not affected by miR-222 (Figure 5B). Transfection efficiency of miR-134 and miR-222 was confirmed by qPCR (Figure 5C). Consistently, transfection of miR-134 mimic inhibited the expression of CD86 mRNA and protein (Figure 5D). Notably, the suppression of protein expression was much more evident than that of mRNA expression. There was no obvious change in the levels of ICAM-1 mRNA and protein in miR-222 mimic transfected cells (Figure 5E). Collectively, these results suggest that miR-134 directly binds to the 3′UTR of CD86 to suppress CD86 expression in B16F10 cells, and miR-222 is unlikely involved in ICAM-1 regulation. Thus, we proceeded to focus on miR-134 in the subsequent experiments.

### miR-134 increased the survival rate of B16F10 cells and inhibited the secretion of IFN-γ and TNF-α

Next, we investigated if HIFU enhanced anti-tumor immunity by inhibiting the negatively regulatory role of miR-134 on CD86 in B16F10 cells. B16F10 cells transfected with miR-134 mimics or negative control (miR-NC) were co-cultured with normal splenic lymphocytes that were pre-stimulated by B16F10. miR-134 efficiently increased the survival rate of B16 cells from 67% to 103% at 24 h and from 44% to 83% at 48 h, compared to that of the miR-NC group (*p* < 0.05, Figure 5F). The levels of IFN-γ and TNF-α in co-cultured supernatant were significantly decreased in cells transfected with miR-134 mimics (*p* < 0.05, Figure 5G). These results suggest that miR-134 mediates inhibition of CD86 in melanoma cells, which leads to suppression of IFN-γ and TNF-α secretion from lymphocytes.

### HIFU treatment promoted anti-tumor immunity of splenic lymphocytes by down-regulating miR-134 level and increasing CD86 expression in B16F10 cells

**Figure 6:**
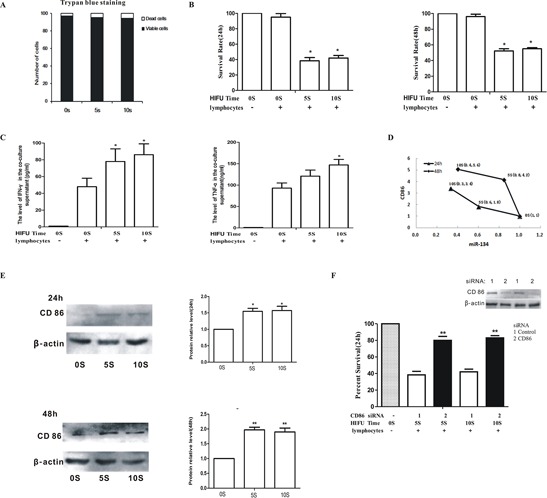
HIFU promoted anti-tumor immunity of splenic lymphocytes by down-regulating miR-134 level and increasing CD86 expression in B16F10 cells **A.** B16F10 cells were exposed to HIFU and cell viability was measured after 6 h by trypan blue staining. **B.** B16F10 cells treated with HIFU were co-cultured with B16F10-activated splenic lymphocytes and the survival rate was determined. **C.** IFN-γ (left panel) and TNF-α (right panel) in co-culture supernatant were measured by ELISA. **D.** miR-134 and CD86 levels were measured by qPCR after HIFU treatment. **E.** The protein levels of CD86 and β-actin were determined by Western blot. **F.** B16F10 cells were exposed with HIFU after transfection with CD86 siRNA and then co-cultured with lymphocytes. The survival rate was measured by MTT. CD86 knockdown was verified by Western blot (Inset). Results are expressed as the mean ± SD. *p < 0.05, **p < 0.01 as compared with the 0 s group.

In order to investigate the mechanism of HIFU-enhanced anti-tumor immune response, B16F10 cells *in vitro* were exposed to HIFU at a moderate dose (4.5 W for 0, 5 and 10 s), and cell viability was measured 6 h post-treatment by trypan blue staining. There was no difference between the three groups (cell survival rate was 97%, 94%, 95%, respectively), indicating the HIFU pretreatment did not directly kill B16F10 cells (Figure 6A). When the cells treated with HIFU were co-cultured with B16F10-activated splenic lymphocytes, the survival rates of control, HIFU 0s, HIFU 5s, and HIFU 10s groups at 24 h were 100%, 95%, 38%, and 42% and at 48 h were 100%, 96%, 55%, and 52%, respectively. Thus, HIFU efficiently decreased the survival rate of B16F10 cells in the co-culture system (*p* < 0.05, Figure 6B). Furthermore, the levels of IFN-γ and TNF-α in the co-culture supernatant were also significantly increased after HIFU pretreatment in an exposure time-dependent manner (*p* < 0.05, Figure 6C). The expression of miR-134 and CD86 was reversely correlated and was in an HIFU exposure time-dependent manner (Figure 6D). In addition, the protein expression of CD86 in B16F10 cells was significantly increased after HIFU pretreatment (*p* < 0.01, Figure 6E). Knockdown of CD86 rescued viability of HIFU-treated B16F10 cells from 40% in control to 80% in the co-culture with splenic lymphocytes (*p* < 0.01, Figure 6F). Taken together, these results imply that HIFU suppresses the expression of miR-134 in melanoma cells to enhance CD86 expression, which enhances anti-tumor immunity by triggering IFN-γ and TNF-α secretion from lymphocytes.

## DISCUSSION

In this study, we demonstrated that HIFU caused necrosis in melanoma tumor tissue to inhibit the growth of the primary tumor, reduced circulating tumor cells and suppressed tumor distant metastasis, and improved mouse survival. HIFU treatment increased the serum levels of IFN-γ and TNF-α, which was in concordance with the therapy effects [20, 21, 27]. This observation was confirmed by *in vitro* studies with the co-culture system, which showed that HIFU enhanced lymphocyte-mediated killing tumor cells through IFN-γ and TNF-α. These results suggest that HIFU induces a systemic anti-tumor immunity.

Our results showed that the expression of miR-134, which is closely associated with immune response, was decreased in HIFU-treated tumor tissues. By bioinformatics analysis, we found that the positive co-stimulatory molecule CD86 is a potential target gene of miR-134. As a positive co-stimulatory molecule, CD86 expressed on the melanoma cell membrane may provide a second signal for T cell activation, which enhances anti-tumor immune response [10]. When the co-stimulatory signal such as CD86 is blocked by miRNA-134, T cells cannot be effectively activated [28]. Lacking co-stimulatory molecules on APC or tumor cells is an important cause of immune tolerance in patients with cancers [10, 11]. Thus, HIFU-induced expression of CD86 may be at least in part contribute to HIFU-enhanced anti-tumor immunity.

We further determined that HIFU promoted CD86 expression through down-regulation of miR-134. By a luciferase reporter assay, we clearly demonstrated that miR-134 directly binds to the 3′UTR of CD86 mRNA to suppress CD86 expression in B16F10 cells. Furthermore, when B16F10 cells were transfected with miR-134 mimics, the expression of CD86 was significantly suppressed mainly at the protein level. Functionally, overexpression of miR-134 in B16F10 cells weakened the cytotoxicity of splenic lymphocytes against B16F10 cells, which was associated with reduced secretion of IFN-γ and TNF-α from co-cultured splenic lymphocytes. Furthermore, under the condition of a moderate dose of HIFU that has little direct cell killing effect, co-culture of lymphocytes efficiently increased HIFU-induced cytotoxicity in B16F10 cells, which was associated with increased secretion of IFN-γ and TNF-α from lymphocytes. Moreover, knockdown of CD86 inhibited the lymphocyte-triggered cytotoxicity in B16F10 cells. All these results support the idea that HIFU enhances anti-tumor immunity through suppressing miR-134 to increase CD86 expression in B16F10 cells that promotes T cell activation. In contrast, HIFU-suppressed miR-222 and enhanced ICAM-1 expression appear to be coincidences, and HIFU modulates ICAM-1 expression is likely through an undetermined mechanism that warrants further studies.

In summary, our results demonstrate that HIFU down-regulates miR-134 to increase CD86 expression, which promotes T cell activation that, in turn, potentiates HIFU's anti-tumor effects. Our study determined a role of miR-134 in HIFU-enhanced anti-tumor immunity, which may be exploited for clinical application of HIFU in cancer therapy. Further studies are needed to investigate whether other miRNAs and other mechanisms are involved in HIFU-enhanced anti-tumor immunity.

## MATERIALS AND METHODS

### Laboratory animals

Female C57BL/6J mice (6–8 weeks old) were purchased from the Animal Center of Chongqing Medical University (CQMU) and housed in the center under pathogen-free conditions before and after HIFU treatment. All animal experiments were approved by the Institutional Animal Care and Use Committee of CQMU.

### Cell line and cell culture

The murine melanoma B16F10 cell line was purchased from ATCC (Manassas, VA) and cultured in RPMI-1640 (HyClone, Logan, UT, USA) supplemented with 10% fetal bovine serum (FBS), 100 U/ml penicillin and 100 μg/ml streptomycin at 37°C in a humidified atmosphere with 5% CO_2_.

### Allograft tumor model, HIFU treatment and measurements

C57BL/6J mice were injected subcutaneously in the right flank with 0.2 ml of a single-cell suspension containing 5 × 10^5^ of B16F10 melanoma cells. The mice were monitored for tumor volume by caliper measurement everyday and tumor volume was calculated by (small diameter) ^2^ × (large diameter)/2. When the large diameter of tumors reached 7–8 mm, the mice were randomly divided into a HIFU group and a sham-HIFU group (55 mice/group).

The Seapostar HIFU System (Chongqing Haifu Technology Co., Chongqing, China) was used for HIFU ablation. The treatment parameter of Seapostar was 9.3 MHz and 4.5 W. Before HIFU treatment, the skin overlying the tumor was epilated and covered with ultrasound transmission gel. The mice in HIFU group received Seapostar treatment, which started from the center of the tumor nodule and expanded gradually towards the boundary with a step size of 1.0 mm. Each treating spot was exposed 10 s and the total exposure time of each tumor nodule was about 120 s. The mice in sham-HIFU group received a sham-HIFU procedure. All HIFU treatments were administered with a safety distance of 1 mm from the tumor margin in order to avoid damage to the adjacent tissues. For studying serum cytokines and spleen cells, a single-cell suspension containing 1 × 10^6^ B16F10 melanoma cells were injected into the tails veins seven days later and sacrificed at day 14 after HIFU treatment, and the serum, tumor tissue and spleen were harvested in each group. For metastasis assay, the mice with tail-vein injection of B16F10 cells were followed up until death. Subcutaneous tumor volume, pulmonary metastasis nodules of tumor and survival rate were determined.

### Hematoxylin-eosin staining

Paraffin-embedded tumor specimens were stained with hematoxylin-eosin (HE), according to the operating instructions (Shanghai Hengyuan Biotechnology Co., Ltd., Shanghai, China).

### Cytotoxicity assay

Spleens from mice in different experimental groups were harvested, dissected, and filtered by stainless steel filter mesh to obtain single cell suspensions. In order to get more pure lymphocytes, the splenic lymphocyte separation medium was used according to the manufacturer′s instructions (Tianjin Haoyang Biological manufacture Co., Ltd., Tianjin, China). The collected lymphocytes (2 × 10^4^) were co-cultured with B16F10 tumor cells (1 × 10^3^) for 24 h, 48 h *in vitro*. The levels of IFN-γ and TNF-α in the co-culture supernatant were measured by ELISA. Cytotoxic activity of lymphocytes against B16F10 cells was measured by MTT assay and the survival rate of B16F10 cells was calculated.

In addition, a part of splenic lymphocytes from normal mice (2 × 10^7^) were pre-stimulated with B16F10 cells (1 × 10^6^) for 5 days in order to activate them *in vitro*, and then were separated and used as effector cells when co-cultured with B16F10 cells which were transfected with miR-134 mimics or treated with HIFU. Cytotoxic activity was detected as described above.

### ELISA

Blood and co-culture supernatant were collected, centrifuged at 1000 *g* × 20 min and stored at −80°C. Specific ELISA kits (Bopei Biotech Co., Ltd., Chongqing, China) were used for measuring IFN-γ and TNF-α, according to the manufacturer's protocol.

### Total RNA isolation, RT-PCR, semi-quantitative RT-PCR and quantitative Real-time PCR

Total RNA from tissues and cultured cells was extracted using TRIzol Reagents (Invitrogen) and cDNA was synthesized from 1 μg of total RNA according to the manufacturer's instructions. cDNA samples synthesized using specific primers for miRNAs and U6(Shanghai Bioligo Biotechnology Co., Ltd., Shanghai, China) were used as template for quantitative real-time PCR (qPCR) for the detection of miRNAs. cDNA samples synthesized using random primer for CD86, ICAM-1 and GAPDH (Shanghai Bioligo Biotechnology Co., Ltd., Shanghai, China) were used as template for semi-quantitative RT-PCR(Semi-PCR). qPCR was performed on the CFX96 real-time PCR detection system from Bio-Rad using SYBR Premix Ex Taq™ II (Takara, Dalian, China). Data were collected and analyzed by the comparative 2^−ΔΔCt^ method with U6 as the control.

### Prediction of the target site for the differentially expressed miRNAs

Target prediction tools PicTar (http://pictar.bio.nyu.edu), miRanda (http://www.microrna.org) and Target Scan (http://www.targetscan.org) were utilized to find out the potential target genes of the differentially expressed miRNAs between the two groups.

### Western blot

Total tissue or cellular extracts were prepared in lysis buffer. Total protein extract (100 μg) was separated by SDS–PAGE and transferred to polyvinylidene difluoride (PVDF) membranes (Millipore, Billerica, MA, USA). The membranes were blocked, washed, and then incubated with specific antibodies against ICAM-1, CD86 or β-actin (Santa Cruz, CA, USA) overnight. After washing, PVDF membrane was incubated with appropriate horseradish peroxidase–conjugated secondary antibody for 1 h at 37°C. Finally, the blots were washed, and then visualized using Bio-Rad chemiluminescence imaging system.

### Construction of p^MIR-REPORT^ luciferase

To construct p^MIR-LUC-3′UTR-CD86^ and p^MIR-LUC-3′UTR-ICAM-1^, CD86 3′UTRs containing miR-134 binding sites and ICAM-1 3′UTR containing miR-222 binding site were generated by Chemical synthesis, and then cloned into downstream of luciferase of pMIR-REPORT™ System (Applied Biosystems/Ambion, Austin TX), respectively. Plasmids containing the 3′UTR mutants (mutation of two nucleotides within the miRNAs binding sites) of CD86 and ICAM-1 were also constructed as control.

### Luciferase reporter assay

B16F10 cells were evenly seeded in a 24-well plate and transfected with pMIR-REPORT constructs (200 ng per well) and Renilla luciferase (pRL-TK Vector, 200 ng per well; Promega, Madison, Wis), plus miR-134, or miR-222 mimics, or miRNA negative control (25 nmol/L, GenePharma, Shanghai, China). Luciferase activity was measured using Single Luciferase Reporter Assay System (Promega Biotechnology Co., Ltd., Beijing, China) 48 h post-transfection, according to the manufacturer′s instructions.

### Transfection and functional assay

B16F10 cells grown to 50–70% confluence in T25 flask were transfected with miRNAs mimics or negative control at a final concentration of 25 nmol/L using Lipofectamine™ 2000 (Invitrogen, Col Anzures, Mexico, CA, USA), according to the instruction. Total protein and RNA were collected after 48 h. One part of the cells transfected with miR-134 were co-cultured with activated splenic lymphocyte for 24 h and 48 h.

### HIFU ablation of B16F10 cells *in vitro*

B16F10 cells cultured *in vitro* were collected and evenly put into three polyvinyl chloride pipe, and then exposed to HIFU for 0 s, 5 s, 10 s at 4.5 W, respectively. After evaluating the survival rate by trypan blue staining, the HIFU-treated cells were cultured in T25 flask, and RNA and protein were isolated at 24 h and 48 h post-treatment. A fraction of B16F10 cells were co-cultured with activated splenic lymphocyte for 24 h, 48 h, followed by MTT assay for measuring cytotoxicity in B16F10 cells.

### RNAi

B16F10 cells (5 × 10^5^/well) seeded in T25 flask were transfected with CD86 siRNA using Lipofectamine™ 2000. As a negative control, non-targeting siRNA duplexes were transfecteded. The knockdown efficiency of siRNA was assessed by Western blot at 48 h posttransfection and the most efficient siRNA was chosen for transfecting B16F10 cells used for HIFU treatment and co-culture with activated splenic lymphocytes.

### Statistical analysis

The results were presented as the mean ± SD. Statistical analyses were performed using SPSS17 statistical software. The survival curves were analyzed by log rank (Mantel-Cox) test, and the others were analyzed by one-way ANOVA and Tukey's Multiple Comparison Test. *p* values lower than 0.05 were considered statistically significant.
